# Health-related quality of life in patients with dyspepsia presenting at the University of Benin Teaching Hospital, Benin City, South-South Nigeria: a cross-sectional study

**DOI:** 10.11604/pamj.2024.47.107.36151

**Published:** 2024-03-05

**Authors:** Oziegbe Egbo, Casmir Omuemu, Edith Okeke, Ojevwe Harrison Egbo, Ndubuisi Mokogwu

**Affiliations:** 1 Department of Internal Medicine, University of Benin Teaching Hospital, Edo State, Nigeria,; 2 Jos University Teaching Hospital, Jos, Plateau State, Nigeria,; 3 Department of Pathology, Edo University, Uzaure, Edo State, Nigeria,; 4 Department of Public Health and Community Medicine, University of Benin Teaching Hospital, Edo State, Nigeria

**Keywords:** Health-related quality of life, dyspepsia, upper gastrointestinal endoscopy, peptic ulcer disease, South-South Nigeria

## Abstract

**Introduction:**

Health-related quality of life (HRQoL) examines the impact of the symptoms of dyspepsia on the daily life of sufferers. There are a few published studies related to HRQoL of persons with dyspepsia in Africa.

**Methods:**

this was a hospital-based cross-sectional study involving 324 dyspeptic patients referred for upper gastrointestinal endoscopy to the University of Benin Teaching Hospitals (UBTH) The ROME IV criteria were used to recruit patients with dyspepsia. The short form Nepean Dyspepsia Index (SF NDI) was used to assess HRQoL in all participants. Upper gastrointestinal endoscopy was performed on all 324 dyspeptic patients.

**Results:**

the mean age of patients was 47.6 ± 15.6 years. Three hundred (92.6%) patients had significantly impaired HRQoL with an SF NDI mean score of 31.3 ± 9.1. Interference with daily activities and eating and drinking subdomains were more impaired than other subdomains of HRQoL (p < 0.001). There was no statistical difference between the impaired HRQoL in patients with functional dyspepsia and organic dyspepsia (p = 0.694). Among patients with organic dyspepsia, those with upper gastrointestinal cancers had significantly worse HRQoL SF NDI mean (sd) scores (39.7 ± 5.9) compared with patients with gastritis, peptic ulcer disease and GERD with (30.3 ± 9.2, 31.5 ± 9.7 and 32.9 ± 7.1 respectively) (p = 0.01).

**Conclusion:**

health-related quality of life is significantly impaired in patients with dyspepsia and those with upper gastrointestinal cancers having overall worse scores. The physical, social and psychological well-being of a majority of patients with dyspepsia in South-South Nigeria is negatively affected by dyspepsia.

## Introduction

Dyspepsia, broadly referred to as chronic pain, centred in the upper abdomen [1], is a symptom complex, which may include epigastric discomfort, bloating, anorexia, early satiety, belching or regurgitation, nausea or heartburn [2]. The ROME IV criteria define dyspepsia as the presence of any of postprandial fullness, early satiation, epigastric pain or epigastric burning, with symptoms present for at least 6 months; with the last three symptoms to be ‘bothersome’ interfering with usual activities [2-5]. Dyspepsia as a symptom cuts across a variety of conditions such as peptic ulcer disease, gastroesophageal reflux disease (GERD), Irritable bowel syndrome, malignancies, pancreatitis, biliary tract disease, motility and vascular disorders [6].

Classification of dyspepsia usually follows investigations which must include upper gastrointestinal endoscopy (UGIE) findings [7]. Patients with detected pathology are classified as organic dyspepsia (OD), those without are said to have functional dyspepsia (FD). Dyspepsia is one of the most common reasons for referral for UGIE in Nigeria [8-12]. The symptoms of dyspepsia as an entity are usually chronic and not life-threatening [13-17]. It is associated with increased health-seeking behaviour, increased medical cost, disruption of daily activities and reduced quality of life of sufferers [18].

Dyspepsia is a common condition that necessitates clinic visits in Nigeria and other parts of the world [15,19,20]. It is responsible for 2-5% of primary care visits, 31.6% of consultations in gastroenterology clinics in Nigeria and 30% in developed countries [20-22]. The prevalence of UD from a global survey including 100 studies in different countries majorly from Northern Europe and South-East Asia, a few from South America, Africa and the Middle East was 21% (with a range of 7% - 35%) [23].

In Africa, the prevalence of UD in Rwanda was found to be 39%, 48.4% in Tanzania and between 26% and 45% from two different studies in Nigeria [24-27]. In a new finding in Nigeria by Nwokediuko *et al*. [28] the prevalence of UD nationwide was 13.09% with the different regions of Nigeria having different prevalence rates [28]. Health-related quality of life is a multi-dimensional concept that includes domains related to physical and mental health perceptions (e.g., energy level, mood) and their correlates including health risks and conditions, functional status, social support and socioeconomic status of an individual [29]. Assessment of HRQoL is best done by asking patients questions through the use of questionnaires which could be generic or disease-specific [30].

Health-related quality of life focuses on the impact of disease on health status as perceived by patients themselves, and its negative influence on physical, social and psychological aspects [31]. It also determines the effect of chronic ailments and their treatment on individuals [29,32]. The majority of patients with dyspepsia have chronic diseases even with treatment [13-15,17,30,33]. There are not too many studies on HRQoL in dyspepsia [34] and a majority of these are in developed countries [23,34]. A study from Africa-Rwanda by Bitwayiki *et al*. [24] revealed impaired HRQoL among dyspeptic health workers. Different studies on dyspepsia regardless of the aetiology, have reported impaired HRQoL in patients [35,36]. This study aimed to ascertain the effect of different aetiology of dyspepsia on HRQoL in patients in South-South Nigeria.

## Methods

**Study design and setting:** this was a cross-sectional study carried out at the University of Benin Teaching Hospital which is an 850-bed Federal government Tertiary Hospital, located in Benin City, Edo State in the South-South geopolitical zone of Nigeria. The hospital serves as a referral center for Edo State and other States such: as Delta, Ondo, Ekiti, Kogi, and Bayelsa. The Endoscopy suite, a part of the Special Investigation Unit of the hospital, has offices, a recovery room, reception, and convenience. There are two functioning endoscopy machines (Aohua VME-2800-used for this study, and the newly acquired Olympus 190 series) among other equipment.

**Study population, sample size, and technique:** dyspeptic patients referred from peripheral hospitals and UBTH were recruited for the study between July 2018 to January 2019.

**Inclusion criteria:** adults 18 years of age and above who must have met the Rome IV criteria 5 for dyspepsia which include: postprandial fullness; early satiety; epigastric pain; epigastric burning with symptoms present for at least 6 months.

**Exclusion criteria:** patients with co-morbidities such as chronic kidney disease and heart failure and those with features of surgical abdomen. e.g. intestinal obstruction or paralytic ileus. Patients who refused to give consent were also excluded from the study.

**Sample size:** a sample size of Three hundred and twenty-four was computed using the Cochran formula for single proportions [37]. Patients who presented for upper GI endoscopy met the inclusion criteria and gave consent were sampled consecutively and recruited for the study until the sample size was completed.

**Data collection and measurement of variables:** data was collected quantitatively with the use of an interviewer-administered tool modified from the short form Nepean Dyspepsia Index (SF NDI). The SFNDI is a validated disease-specific instrument for measuring quality of life in dyspepsia. It is a modification of the 42-item Nepean Dyspepsia Index [38]. The tool has five two-item subscales, with 10 items, and examines the effects of dyspepsia on domains of the patient´s health which include: tension, interference with daily activities, eating and drinking, knowledge and control, work and study. Individual items are measured by 5 point Likert scale, from 1 (not at all), 2 (a little), 3 (moderately), 4 (quite a lot), to 5 (extremely). Each of the 5 subscales measures a minimum of 2 to a maximum of 10, hence giving a minimum total score of 10 and a maximum score of 100. The total overall SF NDI score is calculated by obtaining the mean of the five sub-scale scores. Higher scores indicated poorer HRQoL; patients with scores greater than 15 have been shown to have significantly reduced HRQoL [24,38]. A second questionnaire was used to collect sociodemographic data, social history, drug history, physical examination and abdominal examination findings, endoscopic findings, and documentation of biopsy if taken. The tools were secure till the time for data analysis.

Patients recruited for the study who met the inclusion criteria first had a consent form given to them to read and sign, those who could not read were read to and subsequently signed. The next step was to fill the questionnaire with socio-demographic characteristics., drug, and social history A quick physical examination was carried out on the patients in the endoscopy room and the findings were documented appropriately. Next, the SFNDI questionnaire was filled for each patient after the calculation of HRQoL the third step involved the performance of an upper GI endoscopy. Upper GI endoscopy is a direct examination of the upper gastrointestinal tract from the mouth to the second part of the duodenum. Usually performed by a gastroenterologist after an overnight fast of the patient. Upper gastrointestinal endoscopy was carried out on the patients with an oesophagogastroduodenoscope (AohuaVME-2800, Xenon light source model XLS150-1) and newly acquired Olympus 190 series after they gave consent. Patients for upper GI endoscopy had local anaesthetic (10% xylocaine spray) applied to their oropharynx, put in the left lateral position, and the oesophaogastrodudenoscope was passed from the oropharynx. to the second part of the duodenum under direct vision; after necessary precautions were taken. Findings were observed and recorded. Tissue biopsies from the gastrointestinal tract were obtained (in case of the presence of lesions) with the use of biopsy forceps passed through the biopsy channel of the scope, and preserved in labeled formalin bottles till they were sent to the histopathologist.

**Statistical analysis:** data was analyzed using IBM SPSS version 20.0 software. Categorical data such as age group, sex, and marital status were presented as frequencies and proportions while continuous data like age and HRQoL were represented as mean and standard deviation. Statistical inference was carried out using the independent student´s t-test for differences in mean HRQoL scores among different endoscopic findings in patients with dyspepsia. The level of significance was set at p <0.05. The results are presented using statements and frequency distribution tables.

**Ethical considerations:** all patients had an explanation of the study and only patients who signed prepared consent forms participated in the study. Ethical approval for this study was obtained from the Ethics and Research Committee of the University of Benin Teaching Hospital (chaired by Prof A.N Onunu) Protocol No: ADM/E/A/VOL.VII/1409. All data were kept secure and made available only to the researcher.

## Results

A total of 324 patients with dyspepsia were recruited for the study. The mean ± SD age of study participants was 47.6 ± 15.6 years (Table 1). There were more females (60.8%) than males (39.2%) among the study participants. The majority (72.8%) of patients with dyspepsia had been using proton pump inhibitors while 65.7% were on antacids. The total mean HRQoL scores of patients with dyspepsia was 31.3 ±9.1. Subdomain analysis showed interference with daily activities was worse in dyspeptics with mean scores of 6.8 ± 2.4. Knowledge and control domains were the least affected mean score of 5.6 ± 2.2 in dyspeptics. Using SF NDI, 7.4% of dyspeptic patients in this study had normal HRQoL while 92.6% had impaired HRQoL. Table 2 shows mean SF NDI HRQoL scores in different sub-domains of the patients. Interference with daily activities had the highest mean (sd) HRQoL score of 6.8 ±2.4. Patients with gastric (40.0 ± 7.0) and oesophageal masses (39.0 ± 3.9) had the highest and next highest mean HRQoL SF NDI scores (40.0 ± 7.0 and 39.0 ± 3.9 respectively). The lowest scores were found in patients with duodenitis, gastric polyp, and oesophageal varices (Table 3) Patients with organic dyspepsia had higher mean HRQoL scores 31.4 ± 9.1 compared with patients with functional dyspepsia (30.8 ± 9.4); this was not statistically significant (p=0.694). Both patients with functional and organic dyspepsia had interference with daily activities more affected than other subdomains. Similarly, there was no significant difference in their subdomain scores as shown in Table 4.

**Table 1:** characteristics of study population

Variables	Frequency (n=324)	Percent
**Age group (years)**		
<20	4	1.2
20 – 29	33	10.2
30 – 39	83	25.6
40 – 49	63	19.4
50 – 59	58	17.9
≥60	83	25.6
Mean ± SD age	47.6 ± 15.6 years	
**Gender**		
Male	127	39.2
Female	197	60.8
**Marital status**		
Single	52	16.0
Married	239	73.8
Widowed	24	7.4
Separated/divorced	4	1.2
No response	4	1.2
**Educational status**		
No formal	20	6.2
Primary	46	14.2
Secondary	110	34.1
Tertiary	131	40.6
Postgraduate	16	5.0
**Alcohol use**		
Yes	68	21.0
No	256	79.0
**Smoking**		
Yes	8	2.5
No	316	93.5
**NSAID use**		
Yes	158	48.8
No	166	51.2
**PPI use**		
Yes	236	72.8
No	88	27.2
**Antacid use**		
Yes	213	65.7
No	111	34.3

NSAID: nonsteroidal anti-inflammatory drugs; PPI: Proton-pump inhibitors

**Table 2:** health-related quality of life scores in different domains of patients with dyspepsia

Patients’ sub-domains	SF NDI HRQoL	
	Mean ± SD	Range
Total	31.3 ± 9.1	10.0 - 50.0
Interference with DAs	6.8 ± 2.4	2.0 - 10.0
Eating/drinking	6.6 ± 2.4	2.0 - 10.0
Tension/anxiety	6.3 ± 2.1	2.0 - 10.0
Work/study	6.1 ± 2.5	2.0 - 10.0
Knowledge/control	5.6 ± 2.2	2.0 - 10.0

HRQoL; health-related quality of life, SFNDI: short form Nepean Dyspepsia Index; Das: daily activities

**Table 3:** short form Nepean Dyspepsia Index scores of patients with different clinical conditions from upper gastrointestinal endoscopy

Findings on endoscopy	SF NDI scores (Mean ± SD)
Gastric tumour	40.0 ± 7.0
Oesophageal tumour	39.0 ± 3.9
Oesophageal stenosis	38.0 ± 8.5
Oesophageal candidiasis	36.5 ± 0.7
Duodenal ulcer	35.0 ± 8.3
Hiatal hernia	34.5 ± 5.3
Oesophagitis	32.6 ± 6.9
Bile reflux	31.9 ± 10.0
Normal	30.8 ± 9.4
Gastritis	30.3 ± 9.2
Gastric ulcer	29.9 ± 9.9
Duodenitis	29.2 ± 5.3
Gastric polyp	26.7 ± 14.5
Oesophageal varices	24.6 ± 10.6

SFNDI: short form Nepean Dyspepsia Index

**Table 4:** endoscopic findings and HRQoL scores in patients with organic and functional dyspepsia

Domains	Organic (n =283) Mean ±SD HRQoL scores	Functional (n = 41) Mean ±SD HRQoL scores	T-test statistic	p-value
Total	31.4±9.1	30.8±9.4	0.322	0.694
Tension/anxiety	6.4±2.1	6.0±2.2	0.912	0.362
Interference with DAs	6.8±2.4	6.9±2.2	-0.423	0.672
Eating/drinking	6.6±2.5	6.5±2.2	0.080	0.936
Knowledge/control	5.6±2.1	5.3±2.4	1.003	0.316
Work/study	6.1±2.5	6.0±2.6	0.126	0.900

HRQoL: health-related quality of life; DA: daily activity

The findings on endoscopy were grouped into different disease aetiologies. They comprised gastritis (n=182), peptic ulcer disease (n=77) GERD (n=91), combination (n=69), cancer (n=11) others (n=11). The highest SF NDI values were noted in patients with cancer (mean; 39.7 ± 5.9, p =0.010) who had statistically significantly worse scores for interference with daily activities (mean 9.3±1.2, p = 0.009) and for eating and drinking (mean: 8.7± 1.5, p =0.002) than others (Table 5). The remaining groups and their SF NDI mean scores which represent HRQoL are shown in Table 5. Predictors of impaired HRQoL among study participants were the use of steroids, antacids, and use of proton pump inhibitors, as shown in Table 6.

**Table 5:** comparison of HRQoL scores (using short form Nepean Dyspepsia Index) and endoscopic findings in patients with dyspepsia

Domains (±SD)	Functional dyspepsia	Gastritis	Pud	Gerd	Comb	Cancer	Duodenitis	Others*	F value	p value
Total score	30.8 ± 9.4	30.3 ± 9.2	31.5 ± 9.7	32.9± 7.1	30.8 ± 8.5	39.7 ± 5.9	29.2 ± 5.3	28.2 ± 5.3	2.686	0.010
Tension/anxiety	6.0 ± 2.2	6.2 ± 2.3	6.3 ± 2.2	6.7 ± 1.8	6.1 ± 2.0	7.3 ± 2.1	4.6 ± 1.5	6.0 ± 2.5	1.561	0.145
Interference with daily activities	6.9 ± 2.2	6.5 ± 2.4	6.8 ± 2.5	7.0 ± 2.2	6.6 ± 2.3	9.3 ± 1.2	7.2 ± 2.2	6.1 ± 2.9	2.700	0.009
Eating/drinking	6.5 ± 2.2	6.3 ± 2.5	6.3 ± 2.5	7.1 ± 1.9	6.3 ± 2.3	8.8 ± 1.5	7.4 ± 1.3	5.6 ± 2.5	3.246	0.002
Knowledge/control	5.3 ± 2.4	5.5 ± 2.1	6.1 ± 2.4	5. 6 ± 1.9	5.7 ± 2.4	6.9 ± 1.9	6.2 ± 2.4	4.8 ± 1.6	1.610	0.1303
Work/study	6.0 ± 2.6	5.8 ± 2.6	6.1 ± 2.8	6.5 ± 2.1	6.1 ± 2.5	7.5 ± 2.2	3.8 ± 2.5	5.6 ± 2.6	1.862	0.0739

Others include: Oesophageal candidiasis, varices, achalasia and gastric polyp

**Table 6:** predictors of impaired HRQoL in patients with dyspepsia

Variables	B (regression coefficient)	p-value	Odds ratio	95% CI for OR (Lower- upper)
**Age group**				
>60 years	-0.033	0.910	0.968	0.547 - 1.712
<60 years*			1	
**Gender**				
Female	-0.068	0.802	0.934	0.551 - 1.586
Male*			1	
**Alcohol use**				
Yes	0.470	0.127	1.599	0.875 - 2.923
No*			1	
**Smoking**				
Yes	-1.528	0.179	0.217	0.023 - 2.017
No*				
**Nonsteroidal anti-inflammatory drugs**				
Yes	-0.076	0.769	0.927	0.559 - 1.537
No*			1	
**Steroids**				
Yes	1.254	0.001	3.505	1.434 - 8.567
No*			1	
**Antacids**				
Yes	-0.888	0.006	0.412	0.243 - 0.699
No			1	
**Proton-pump inhibitors**				
Yes	-1.839	0.001	0.159	0.092 - 0.274
No			1	

* Reference category; R2 (coefficient of determination) = 23.3% to 33.2%

## Discussion

This study aimed to determine the HRQoL in patients with different aetiologies of dyspepsia after having an upper gastrointestinal endoscopy. To our knowledge for now, this research seems to be the first determining the HRQoL in patients with dyspepsia in Nigeria, we found out from this study that HRQoL was impaired in a significant number of patients with dyspepsia regardless of the underlying aetiology.

In this study the HRQoL of patients was assessed using a specific instrument -the short form Nepean Dyspepsia Index (SF NDI) on five domains, viz: tension/anxiety, interference with daily activities, eating/drinking, knowledge/control, work/study. It was found that a significantly high proportion of patients with dyspepsia had impaired HRQoL, with all domains affected, however, Interference with daily activities was most affected. The patients with dyspepsia had eating and drinking affected negatively, had increased tension and anxiety, and also reduced attendance and productivity at work as the case may be, with these findings, we could imply that dyspepsia affected the overall physical, mental, and psychological well-being of most of the sufferers, and also worsens their economic States as it affected most of the patients´ work negatively. Our study was consistent with that of Bitwayiki *et al*. to assess the prevalence of dyspepsia and its impact on health-related quality of life among hospital workers in Rwanda [24]. The workers in Rwanda however did not have Upper GI endoscopy, instead Short Form Leeds dyspepsia questionnaire was used to determine the presence and frequency of dyspepsia, following which the SF NDI was applied to assess HRQoL.

Findings from other studies published between 2010 and 2017 also demonstrated impaired HRQoL among patients with dyspepsia. These studies utilized generic instruments such as SF 36, and SF 12QOL amongst others [36,39-41]. Generic instruments can broadly access HRQoL in patients with dyspepsia however, it is not very sensitive to changes in specific symptoms in patients with dyspepsia, hence the use of SF NDI a specific instrument in this study [42]. Among patients with dyspepsia in our study, Interference with daily activities and, eating and drinking subdomains of HRQoL were most affected, this means that the majority of patients with dyspepsia had their daily activities such as work, religious, and social activities disrupted by symptoms. Reduction in eating and drinking could contribute to malnutrition in extreme cases. A study from Malaysia [43] is however in contrast with our study and observed Interference with daily activities and eating and drinking subdomains of HRQoL were least affected in patients with dyspepsia. The majority of patients from the study in Malaysia had a high prevalence of functional dyspepsia, this and the difference in diet may be responsible for the difference in the subdomain of HRQoL affected.

In this study, there was interestingly no significant difference in HRQoL between patients with functional dyspepsia and organic dyspepsia, demonstrating that the presence or absence of lesions on upper gastrointestinal tract endoscopy did not have a significant change in the HRQoL amongst the patients with dyspepsia. This may be because studies have shown that mental distress (tension) which is a component of HRQoL is common in patients with functional and organic dyspepsia and there was no difference between the risk of mental distress between the two aetiologies of dyspepsia [44]. This is similar to a study in Saudi Arabia that, found no statistically significant difference in overall HRQoL indices between patients with GERD (a cause of organic dyspepsia) and functional dyspepsia; the mean scores showed that people with functional dyspepsia were more affected than people with GERD a cause of organic dyspepsia and this was mostly in psychological component. The difference in this was possibly because the patients could not attribute their ailment to a specific medical or organic disease leading to increased levels of mental stress [39].

In this study, the majority of patients were classified as having organic dyspepsia after upper gastrointestinal endoscopy. In these patients with organic dyspepsia, those with upper gastrointestinal malignancies had the worse HRQoL compared with the other aetiologies. Interference with daily activities and eating and drinking subdomains were most affected; this may be attributable to any or all of the weakness and pains preventing the patients from carrying out their regular activities and severe nausea, anorexia, vomiting, and abdominal pains which are associated with oesophageal and gastric malignancies affecting normal feeding. In the literature reviewed, there was a paucity of studies that compared the quality of life among patients with dyspepsia and upper GI malignancies with other causes of organic dyspepsia using SF NDI. European Organisation for Research and Treatment of Cancer core QOL questionnaire (EORTC QOL) QLQ C30 are validated tool for assessing QOL in patients with upper GI malignancies [45]. Lagergren *et al*. [46] in a study of the clinical and psychometric validation of the EORTC CQLQ 30, revealed that people who had surgery for oesophageal cancer had poor quality of life which became worse than baseline after surgery.

Among patients with non-malignant causes of organic dyspepsia like gastritis, peptic ulcer disease GERD, etc., patients with GERD had worse HRQoL, with eating and drinking subdomains of HRQoL most affected. GERD is known to affect HRQoL by disruption of sleep due to reflux symptoms, and prolonged symptoms with buildup of anxiety; GERD has extraesophageal symptoms such as laryngitis, asthma, otitis media, dental erosions, etc. and these extra oesophageal symptoms are combined with the symptoms of GERD to worsen HRQoL. A study by Wikulnd I showed impaired HRQoL in patients with GERD compared with the general population; with the impairment similar to patients with chronic medical conditions such as congestive cardiac failure [47].

This study reveals that patients with peptic ulcer disease generally had higher SF NDI scores and worse HRQoL than patients with gastritis; and amongst patients with peptic ulcer disease, those with duodenal ulcers had significantly worse HRQoL compared to patients with gastric ulcer. The reason for this might be related to the initial relief of abdominal pain in patients with duodenal ulcers while eating but pain recurring 2 to 3 hours after food, particularly at night and disturbing sleep [48].

In this study even though more females were investigated for dyspepsia, gender was not a predictor of impaired HRQoL in patients with dyspepsia. Age was also not a predictor of impaired HRQoL among patients with dyspepsia. However, a study by Hantoro *et al*. [40] among patients with FD showed that increasing age and female gender were associated with worsening HRQoL. This study also shows that those who used steroids were likely to have impaired HRQoL while those who used antacids and PPIs were less likely to have impaired HRQoL. This could be attributed to the healing effects of PPIs on the aetiologies of dyspepsia with a reduction in pain and discomfort.

This study is the first of its kind in Nigeria and one of the few cases in Africa where dyspepsia is classified with the use of upper GI endoscopy with histology and not just the use of questionnaires when assessing the HRQoL in patients with dyspepsia. The different sub-domains of the HRQoL of the study participants were based on self-reports and may be difficult to verify. The authors, however, encouraged the patients to be accurate as possible.

## Conclusion

Organic dyspepsia is more prevalent in patients with dyspepsia in Benin City and gastritis was the most common finding on upper gastrointestinal endoscopy. Patients with dyspepsia whether functional or organic, have significantly impaired HRQoL. Upper gastrointestinal cancers are possible findings following upper GI endoscopy and its sufferers have overall worse HRQoL. It was found that a significantly high proportion of patients with dyspepsia had impaired HRQoL, with all domains affected, with Interference with daily activities being most affected. we could imply that dyspepsia affects the overall physical, mental, and psychological well-being of most of the sufferers, and also worsens their economic states as their daily activities which contribute to daily earnings were affected by symptoms. These impairments in HRQoL in dyspepsia are comparable to those of chronic medical conditions such as heart failure, hence the need for proper attention on the symptoms of dyspepsia, to improve the physical, mental, and psychological state of patients. Upper GI endoscopy should be recommended for patients with dyspepsia who meet criteria as serious conditions like malignancies could be an underlying aetiology, early detection may improve outcomes in such patients.

### What is known about this topic

- *Information on HRQoL in dyspepsia is mainly in developed countries;*
- *Dyspepsia impairs the health-related quality of life of sufferers affecting the social-psychological function of sufferers;*
- *Health-related quality of life in dyspepsia is best accessed with the use of instruments like the short form Nepean Dyspepsia Index, for objectivity*.

### What this study adds

- *A new study of health-related quality of life in sub-Saharan Africa in patients who have dyspepsia, can be referenced, instead of sitting studies from developed countries;*
- *Organic dyspepsia is the major cause of dyspepsia in South-South Nigeria and it is important to have upper GI endoscopy as serious medical conditions like malignancies could be the underlying aetiology;*
- *Health-related quality of life is seriously affected in patients with dyspepsia in South-South Nigeria*.
